# A case study to investigate the effects of Chlorhexidine mouthwash on serum cytokines levels in children with Asthma

**DOI:** 10.1186/s13104-024-06831-7

**Published:** 2024-06-26

**Authors:** Saeed Sadr, Sahar Eslaminezhad, Entezar Mehrabi Nasab, Poonam Arora, Bahram Yavari, Hadi Khodabandehloo, Davood Jafari, Mohammad Borji, Seyyed Shamsadin Athari

**Affiliations:** 1 Department of Pediatric Pulmonology, Mofid Children’s Hospital, Shahid Beheshti University of Medical Science, Tehran, Iran; 2 Department of Molecular and Cellular Biology, Islamic Azad University of Kazerun, Kazerun, Iran; 3 Department of Cardiology, School of Medicine, Tehran Heart Center, Tehran University of Medical Sciences, Tehran, Iran; 4 Department of Cardiology, School of Medicine, Valiasr Hospital, Zanjan University of Medical Sciences, Zanjan, Iran; 5 Department of Pharmacognosy and Phytochemistry, SGT College of Pharmacy, SGT University, Gurugram, Haryana India; 6 Department of Medical Biotechnology, School of Advanced Medical Sciences & Technologies, Hamadan University of Medical Sciences, Hamadan, Iran; 7 Department of Clinical Biochemistry, School of Medicine, Zanjan University of Medical Sciences, Zanjan, Iran; 8 Department of Immunology, School of Medicine, Zanjan University of Medical Sciences, Zanjan, Iran

**Keywords:** Chlorhexidine, Interleukin, Asthma, Allergy

## Abstract

Asthma is an airways inflammatory disease and the most common chronic disease of childhood, which causes most hospital visits and placing a heavy financial burden on families and communities. Interleukins 4, 5 and 13, play a central role in the pathogenesis of asthma. Given the importance of oral hygiene in asthmatic patients and IL-4 and 5 are involved in the inflammatory process of periodontitis, the effect of chlorhexidine as mouthwash on asthma attacks in children on serum cytokines is necessary. In this study, 375 children with asthma were divided into two groups using or non-using chlorhexidine. Blood samples were taken and cytokines were measured by ELISA. From 375 patients, 17 patients were excluded. In this study, 171 males and 187 females participated and there were 180 patients in asthma group and 178 patients in asthma/Chlorhexidine group. The levels of IL-4, IL-5 and IL-13 had no significant difference (*p* > 0.05) between Asthma and Asthma/Chlorhexidine groups. Using chlorhexidine as mouthwash in children with asthma had no effect on the type 2 cytokines and may not trigger an asthma attack via allergo-inflammatory mechanism.

## Introduction

Asthma is a chronic inflammatory disease of the airways that is associated with bronchoconstriction, inflammation, dyspnea and is characterized by clinical symptoms of coughing, wheezing, and shortness of breath triggered by to endogenous or exogenous stimuli [1, 2]. The global prevalence of asthma is estimated to be more than 350 million people worldwide, with most cases of asthma occurring in childhood [3]. In the last year, the prevalence of wheezing in Iranian children, aged 6 to 7 years and 13 to 14 years was found as 7.6 and 10.7%, respectively [4] and more than 30% of children were hospitalized in the United States due to asthma [3, 4], which is estimated to be the most common cause of hospitalization of children in North America [1, 2], causing a heavy financial burden on the family and the community. It is also the leading cause of school absenteeism and emergency department and hospital admissions in children. Therefore, the country and the world urgently need to pay more attention to this problem [5–9].

In asthma, the pathophysiology of disease is driven by T cell related immune response where Th2 cells play a key role in mediating airway hyper-reposnsiveness and airway inflammation. Activated lymphocytes release specific cytokines, including interleukin-4 (IL-4), interleukin-5 (IL-5), interleukin-6 (IL-6), interleukin-9 (IL-9), and interleukin-13 (IL-13) [5–7, 9, 10]. On the other hand, interleukins 4, 5 and 13, as the main cytokines in asthma attacks and eosinophil activation, play a central role in the pathogenesis of asthma. Children with chronic medical disorders such as asthma who require long-term medication are more susceptible to dental disease due to the use of sugar syrups, corticosteroids and sedatives that reduce salivation, and therefore, in mild and moderate cases of asthma, decreased salivary flow, increased caries and gingivitis, mucosal changes associated with chronic oral respiration and maxillary abnormalities, and periodontal disease can be seen alongside general symptoms [1, 3, 10–12].

In periodontal diseases, subgingival bacteria trigger release pro-inflammatory mediators in the periodontal tissues contributing to more progression. Presence of increased levels of cytokines including interleukin IL-4 has been demonstrated in periodontitis, which may further exacerbate the inflammatory state in asthma [13]. In dentistry, the most popular chemical for oral hygiene is chlorhexidine. Its antimicrobial and anticoagulant properties make it an excellent bacteriostatic and bactericidal agent. However, it has certain side effects such as brown discoloration of the teeth, erosion of the oral mucosa and a bitter taste [14].

Given the importance of oral hygiene in asthmatic patients and the fact that interleukins 4 and 5 are involved in the inflammatory process of periodontitis, it is likely that chlorhexidine could help reduce progression of asthma. Since, very few studies have been conducted related to evaluating role of chlorhexidine mouthwash on exacerbation of asthma in children and finding its significant clinical importance in modern dentistry, in the present study, we decided to investigate the effects of chlorhexidine mouthwash (the gold standard for maintaining oral hygiene) on serum interleukins 4, 5 and 13 in children.

## Materials and methods

This study was performed on children with asthma who were referred to the Asthma and Allergy Clinic of Mofid Children’s Hospital in Tehran. In this study, 375 children with asthma who met all the clinical and paraclinical diagnostic criteria for asthma, were included in the study. A patient checklist was designed to record the patient’s demographic information of patients and all their medical information were entered based on the registry system and according to the clinical guideline. Prior to the start of the project, all patients were fully informed about the blood collection process and the project methodology. All samples were entered with personal and written consent. The present study is a case study and researchers do not interfere in the use or non-use of mouthwash.

Experimental protocol; patients were divided into two groups, first, case group and second, control group. The control group was asthmatic group and the case group was asthmatic group that used chlorhexidine as mouthwash. The researchers did not perform any trial on the patients and only grouped based on their previous information on whether or not to use mouthwash. In fact, the criterion for grouping is the history of mouthwash use in asthmatic patients, and a case-control study was performed. Therefore, the researchers did not intervene in the use of chlorhexidine mouthwash by asthma patients, viz., and group of people with asthma who used chlorhexidine mouthwash with another group of participants who had asthma and did not use chlorhexidine mouthwash, participated in this study. The control group used chlorhexidine mouthwash twice a day for two weeks, but the control group did not use mouthwash. Both groups were matched in terms of confounders. All patients with immunodeficiency, immunosuppressive therapy, infections, inflammatory diseases, malnutrition, and other problems were excluded from the study. Finally, 5 cc blood samples were collected from the patients and stored at -70 °C. After starting the assay, quality control and assurance of all samples were measured together by ELISA method.

Data were entered into SPSS software version 22 and assigned after allocating the appropriate codes. Data of the both groups were analyzed using t-test. The significance level for all tests was less than 0.05.

## Result

**Fig. 1:**
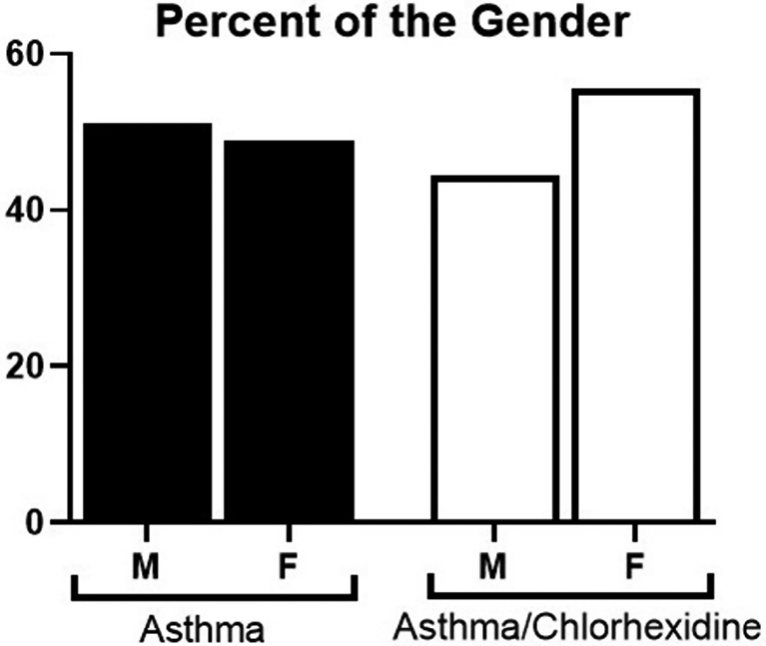
The percentage of sexes in the studied groups (asthma and asthma/chlorhexidine groups

From 375 asthmatic patients, 17 patients were excluded and 358 patients were remained that were 180 patients in asthma group and 178 patients in asthma/Chlorhexidine group (Table 1). In this study, 171 males and 187 females participated (Fig. 1).

**Table 1:** A total of 358 patients with asthma were included in two study groups (asthma and asthma/chlorhexidine groups) and included in two male and female gender in 4 age subclasses

		M 1–3	M 4–6	M 7–9	M 10–12	F 1–3	F 4–6	F 7–9	F 10–12
Asthma		17	36	8	31	9	26	43	10
Asthma/Chlorhexidine		0	17	29	33	1	35	31	32
Total	358	171				187			
Asthma		M: 92	F: 88	Total: 180					
Asthma/Chlorhexidine		M: 79	F: 99	Total: 178					

**Fig. 2:**
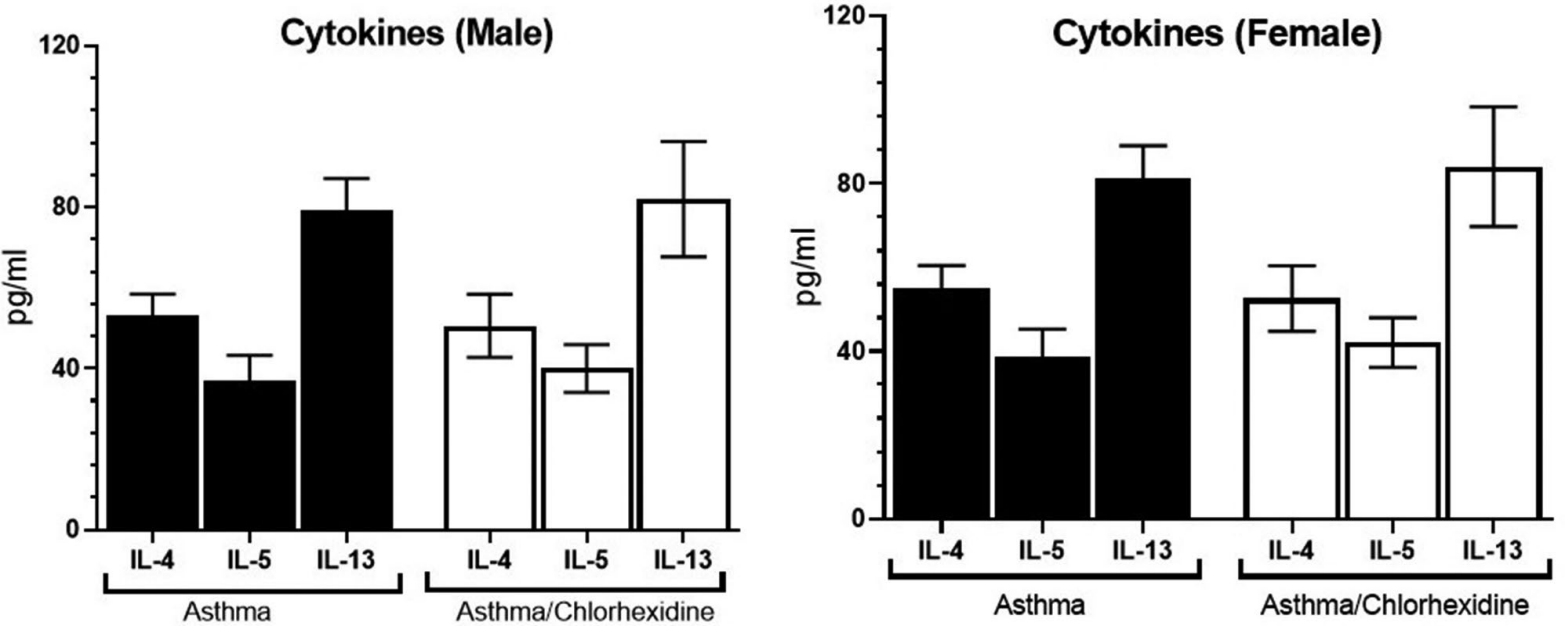
The levels of IL-4, IL-5 and IL-13 were measured in the serum of asthma and asthma/chlorhexidine groups in two male and female sexes. There was no significant difference between asthma and asthma/chlorhexidine groups (*p* > 0.05) and also between male and female sexes (*p* > 0.05)

The levels of IL-4, IL-5 and IL-13 had no significant difference (*p* > 0.05) between asthma (54.17 ± 5.28, 37.82 ± 6.42, and 80.14 ± 7.9 pg/mL respectively) and asthma/chlorhexidine (51.56 ± 7.82, 41.03 ± 5.92 and 82.99 ± 14.28 pg/mL respectively) groups (Fig. 2).

Male asthma/chlorhexidine patients had no significant changes (*p* > 0.05) compared to male asthma patients in IL-4, 5 and 13 levels. Also, female asthma/chlorhexidine patients had no significant changes (*p* > 0.05) in cytokine levels as compared to female asthma patients.

## Discussion

Asthma is a chronic inflammatory disease of the airways that is exacerbated in people who are allergic to certain environmental factors. This inflammatory phenomenon obstructs the airways and causes the main symptoms of the disease and affects the interaction of cells such as lymphocytes and their products such as cytokines on the development and progression of the disease [15, 16]. Asthma is also a disease with a second type cytokine pattern, in which the levels of cytokines in this group, especially IL-4 and IL-5, increase in response to allergens, leading to the inflammatory state of asthma [15, 16]. Reddy et al. (2011) examined 205 children up to the age of 8 with asthma and found that the prevalence of tooth decay in asthmatic children was significantly higher than in healthy children [17]. In a study by Ersin et al. (2009) on 133 children with asthma and 136 healthy children aged 6 to 11 with the same age and socioeconomic status, they concluded that asthma and related medications reduced pH and salivary flow and this increases the risk of dental caries in asthmatic children. They also found that the duration of illness and medication also had a significant effect on increasing caries in asthmatic children [18]. In the present study, the relationship between dental caries and asthma was not investigated and the changes of type 2 cytokines in asthmatic and asthmatic patients with using chlorhexidine were investigated.

In 2018, Stavroullakis et al. investigated the effect of two compounds, chlorhexidine diacetate (CHX) and epigallocatechin-gallate (EGCG) on the levels of cytokines produced by odontoblast-like cells (MDPC-23). Total protein concentrations and 23 cytokines were measured after 48 h. The results showed that CHX and EGCG significantly increased the secretion of interleukins 1, 10, 12, 6, macrophage inflammatory protein (MIP-1) and INF-γ. CHX increased IL-4 and RANTES, and both CHX and EGCG significantly reduced IL-17 secretion [19]. In the study, it was found that the use of chlorhexidine as a mouthwash in asthmatic patients had no effect on changes in type 2 cytokines levels. Therefore, levels of interleukin 4, 5, and 13 in asthmatic patients with chlorhexidine using did not differ significantly from the levels of these cytokines in asthmatic patients without chlorhexidine using.

Hygor et al. in 2019 had study on the effect of component gel chlorhexidine on the expression of pro-inflammatory genes (COX-2, NOS-2, INF-γ, OSCAR and MYD88) and anti-inflammatory genes (IL-10, IL-4 and TGFβ1) in rat gingival tissues. Result showed that the gene expression profile in gingival tissues with periodontitis was not significantly different between the two treatments. This study showed that treatment in the periodontitis model significantly reduced the expression of genes (COX-2, NOS-2, INF-γ, OSCAR and TGFβ). There was no significant difference between the treated groups and the healthy group in IL-4 level [20]. In 2009, Türkoğlu et al. selected fifty patients with gingivitis to evaluate the effectiveness of chlorhexidine mouthwash on daily plaque control in gingivitis and randomly divided them into CHX or placebo groups. The values of IL-1a, IL-1b, IL-1Ra and IL-8 were determined. The results of their work showed that CHX mouthwash can be useful in the management of plaque-related gingivitis, although it has no significant effect on the level of cytokines [21]. No gender or age differences were observed in this study. This study was also conducted in asthmatic children using chlorhexidine mouthwash and the amount of type 2 cytokines was measured and compared with the serum cytokines of asthmatic children who did not use chlorhexidine mouthwash. In the present study, healthy individuals without a history of asthma in the control group and the chlorhexidine mouthwash group were not used.

According to result, it was observed that using chlorhexidine as mouthwash in children with asthma had no effect on the type 2 cytokines levels (IL-4, IL-5 and IL-13) in serum. This may be related to the method of use of chlorhexidine mouthwash, which is used as a topical disinfectant rather than by oral or injection. Also, unsafe administration of chlorhexidine as mouthwash may lead to side effects and may increase allergic type 2 cytokines, initiate allergic response and exacerbate allergic asthma attack. But using chlorhexidine as mouthwash can be safe and may not trigger an asthma attack via allergo-inflammatory mechanism and type 2 cytokines. Finally, it was suggested that this study is repeated in large scale and in large number of asthmatic patients and also, in healthy peoples without asthma and allergic problems. We had several limitation in this study that include; we could not study the effects of Chlorhexidine mouthwash on levels of serum cytokines in different ages. Also, we could not study the effects of Chlorhexidine mouthwash on all serum cytokines. We could not evaluate the teeth brushing effects on serum cytokines levels and also, we had limitation in study the effects of Chlorhexidine mouthwash using by parents on levels of serum cytokines on their children.

## Data Availability

No datasets were generated or analysed during the current study.
